# Selective detection of Ochratoxin B in food by fluorescent sensor based on 2-mercapto-5-benzimidazole carboxylic acid-capped CdSe quantum dots

**DOI:** 10.1016/j.fochx.2026.103770

**Published:** 2026-03-17

**Authors:** Wenxin Chen, Jiahao Fu, Yaqi Liu, Yao Fan, Haiyan Fu, Yuanbin She

**Affiliations:** a State Key Laboratory of Green Chemical Synthesis and Conversion, College of Chemical Engineering, Zhejiang University of Technology, Hangzhou 310032, PR China; b The Modernization Engineering Technology Research Center of Ethnic Minority Medicine of Hubei Province, School of Pharmaceutical Sciences, South-Central University for Nationalities, Wuhan 430074, PR China

**Keywords:** Ochratoxin B detection, Imidazolyl modification, Quantum dots, Fluorescence sensor

## Abstract

Ochratoxin B (OTB) that widely presented in food has significant cytotoxicity even at low concentration levels. Herein, a novel fluorescent sensor based on the 2-mercapto-5-benzimidazole carboxylic acid (MBI)-capped CdSe quantum dots (QDs) was established for the selective detection of OTB in food. Specifically, the synergistic effect of electrostatic interaction and intermolecular hydrogen bonds between MBI and OTB provided a direct and sensitive fluorescence signal that enables efficient recognition of OTB from other mycotoxins, even OTA with similar structures. Excellent linear detection performance for OTB is exhibited with a range from 0.037 ng/mL to 48.01 ng/mL and a detection limit of 0.016 ng/mL. Furthermore, our developed method exhibits good anti-interference stability against contaminants such as mycotoxins, inorganic salts and amino acids. Most importantly, the sensor demonstrates high sensitivity in real food samples with the recovery ranges from 96.51% to 104.7%, with an RSD of less than 2.67%.

## Highlights

• A novel fluorescent sensor based on the MBI-capped CdSe QDs was established.
• The sensor enables efficient recognition of OTB from other mycotoxins, even OTA.
• The detection performance for OTB exhibited with wide range and low detection limit.
• The sensor demonstrates high sensitivity in real food samples.

## 1 Introduction

As one of the key factors, mycotoxin contamination has always been a focus of attention in the global food safety field. Among them, Ochratoxins not only pose a risk of multi-toxin synergy but also have acute and chronic toxicological effects at trace levels (Frisvad et al., 2019; Wu et al., 2024). Researchers have devoted most of their attention to ochratoxin A (OTA) nowadays due to its higher toxicity. For other types such as ochratoxin B (OTB), there is insufficient research on their detection. OTB and OTA have a high degree of similarity in chemical structure, both belonging to isocoumarin compounds, with only a difference of one chlorine atom in the side chain of phenylalanine (Ben Miri et al., 2024; Xu et al., 2022). As analogues of OTA, OTB usually coexists with OTA, and these OTs can transform into each other even at very low concentrations (Ben Miri et al., 2024; Peng et al., 2022; Peng et al., 2023; Wei et al., 2023). On the other hand, OTB also shows obvious renal toxicity, hepatotoxicity, immunotoxicity, teratogenic properties, and genotoxic effects (Qileng et al., 2018; Tang et al., 2019). When first tested on animals, the oral LD50 value of ochratoxin B (OTB) was approximately 54 mg/kg, showing similar acute cytotoxicity to OTA. Additionally, OTB also demonstrated immunotoxicity in the study (Casu et al., 2024; Peng et al., 2022). However, compared with OTA, the detection of OTB has not received sufficient attention for a long time. The food safety supervision systems of various countries mainly focus on the control of OTA (Janik et al., 2021; Liang et al., 2025). This regulatory gap has led to the hidden pollution risks of OTB being largely ignored, while the potential harm of OTB entering the human body through the food chain objectively exists (Chandravarnan et al., 2024; Frisvad et al., 2019; Nekrasov et al., 2022; Veenuttranon et al., 2024). Therefore, establishing a precise and efficient detection method for OTB content is of vital significance for in-depth research on the potential threat of its hidden pollution to the food chain and for ensuring the health and safety of consumers.

Traditional mycotoxin detection techniques for ochratoxins include enzyme-linked immunosorbent assay (ELISA), chromatography-mass spectrometry (LC-MS/MS), etc. (Alford & Mishael, 2023; Chandravarnan et al., 2022; Janik et al., 2021; Wu et al., 2024; Yang et al., 2024). The ELISA method features a standardized operation process, low instrument cost, and a short detection cycle, making it suitable for the preliminary contamination assessment of large-scale samples (G. Wang et al., 2025; Wu et al., 2024; Xie et al., 2024). However, in OTB detection, there is a challenge of cross-reaction interference. Due to the fact that OTB and OTA only have the difference of chlorine atoms in the phenylalanine side chain in their chemical structures, this method faces the problem that conventional antibodies are prone to cross-recognition with OTA in OTB detection, leading to quantitative deviations (Alford et al., 2023; Ben Miri et al., 2024; Janik et al., 2021). Liquid chromatography-tandem mass spectrometry (LC-MS/MS) is suitable for the detection of trace OTB with complex components (G. Wang et al., 2025; Yang et al., 2024). However, its detection cycle is long, the sample pretreatment steps are cumbersome, and the instruments are expensive. In addition, the electrochemical (ECL) sensors optimized by nanomaterials have enhanced detection sensitivity due to their excellent optical performance and surface modifiable properties, demonstrating great application potential (Veenuttranon et al., 2024). However, at present, their application is only used for OTA detection, and its application scope has not yet been expanded to OTB detection. Otherwise, fluorescence sensors are an important technique for the detection of ochratoxin especially OTB. Compared with traditional detection methods that are difficult to meet the demand for rapid screening, this method does not require complex labeling, is easy to operate, and has a relatively high sensitivity (Song et al., 2021; Zhang et al., 2024). The fluorescence nano sensor established by Su achieved ultra-sensitive detection of OTA (detection limit 5 pg/mL, linear range 5–5000 pg/mL), and its high specificity was verified through cross-reaction between OTB and OTC (Su et al., 2022). The multi-colorimetric to ratio fluorescence sensor developed by Zhu has a detection limit of 0.56 pg/mL for OTA, with a linear range of less than 1 ng/L-10 μg/L (Zhu et al., 2021). According to the existing literature, there have been no reports on fluorescence sensors for OTB.

Quantum dots (QDs), as a new type of nano-fluorescent material, have become a key material for OTs detection due to their high fluorescence quantum yield, narrow symmetrical emission peak and adjustable particle size fluorescence characteristics (Ding et al., 2021; Vartanian, 2023). Jia has developed a dual-color FRET sensor, which uses carbon dots (CDs) and CdZnTe quantum dots (QDs) as dual-nanometer donors and MoS₂ nanosheets as a single acceptant to simultaneously detect aflatoxin B₁ (AFB₁) and OTA (Jia et al., 2022); Jin used amino-thiol modified dendritic mesoporous silica (A-T-DMSNs) loaded with multicolor QDs as the signal probe to achieve simultaneous detection of four toxins: OTA, AFB₁, fumonisin B₁ (FB₁), and zearalenone (ZEN) (Jin et al., 2024); Qian then constructed a CdSe@CdS QDs sensor, but it was only used for OTA detection(Qian et al., 2020). However, no literature has reported the use of quantum dots for OTB detection yet.

**Scheme 1:**
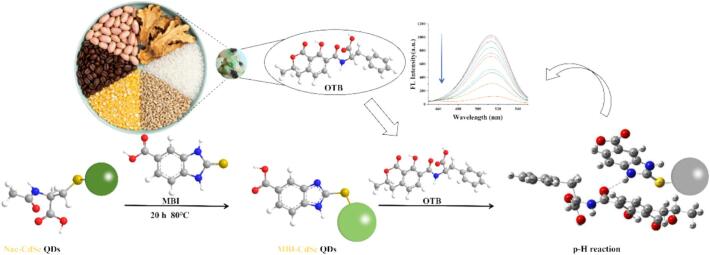
Schematic diagram of imidazolium-based quantum dots for the detection of OTB.

The QDs modified by the imidazole group have been widely applied in fields such as biosensing, chemical sensing and environmental monitoring due to their tunable fluorescence properties and diverse binding sites(Cheng et al., 2021; Molinaro et al., 2020; Movellan et al., 2020; G.-X. Yang et al., 2022; J. Yang et al., 2022), and particularly had the potential to enhance the affinity for OTB (Kim & Nimse, 2025; Ni et al., 2018; J. Wang et al., 2016). Herein, a novel 2-Mercapto-5- benzimidazole carboxylic acid (MBI for short) -modified cadmium selenide quantum dots (CdSe QDs) were successfully designed and synthesized (Scheme 1). Imidazole groups are introduced into the surface of quantum dots, not only providing abundant binding sites for the detection of OTB, but also achieving the efficient conversion of recognition signals to optical signals. The excellent linear detection performance for OTB is exhibited, ranging from 0.037 ng/mL^−1^ to 48.01 ng/mL^−1^, with a detection limit of 0.016 ng/mL^−1^. The fluorescence sensing platform based on MBI-modified CdSe QDs not only provides a new strategy for the detection of mycotoxins, but also has the advantages of rapidity, low cost and high sensitivity, and is expected to play an important role in food safety monitoring in the future.

## 2 Experimental section

### 2.1 Chemicals

Ochratoxin B (OTB), ochratoxin A (OTA), aflatoxin B_1_ (AFB_1_), deoxynojirimycin (DON), fumonisin B_1_ (FB_1_), fumonisin B_2_ (FB_2_), T-2 toxin (T-2), α-ZEA, zearalen-10 (ZEA), and zearalen-3 (ZEN) were purchased from Yuan Ye Biotechnology Co., Ltd. CaCl_2_, FeCl_3_, HCl, KCl, MgSO_4_, NaCl, and NaOH were procured from Sinopharm Group Chemical Reagent Co., Ltd. Selenium powder, and sodium borohydride were supplied by Macleung Biochemical Technology Co., Ltd. Chromium chloride (CdCl_2_·2.5H_2_O), *N*-acetyl-L-cysteine (NAC), arginine (Arg), histidine (His), isoleucine (Ile), leucine (Leu), lysine (Lys), 2-mercapto-5-benzimidazole carboxylic acid (MBI), phenylalanine (Phe), proline (Pro), serine (Ser), and tyrosine (Tyr) were obtained from Aladdin Biochemical Technology Co., Ltd. All chemical products are of analytical purity.

### 2.2 Synthesis of MBI-CdSe quantum dots

First, CdSe quantum dots were synthesized via the hydrothermal method. MBI (0.0046 g) was added to 80 mL of CdSe quantum dot solution. The reaction was conducted for 20 h under 80 °C conditions. After cooling to room temperature, an orange-yellow fluorescent MBI-CdSe quantum dot solution was obtained. The MBI-CdSe quantum dot mixture was then added to ethanol at a 1:2 volume ratio and centrifuged at 10,000 rpm for 15 min. The supernatant was discarded, leaving the precipitate as the successfully prepared MBI-CdSe quantum dots, which were stored in a 4 °C refrigerator for later use. The final product was dispersed in water before application.

### 2.3 Quantitative determination of ochratoxin B by MBI-CdSe fluorescence spectrometry

Preparation of actual samples: Accurately weigh 0.1 g of actual sample powders (rice, corn, Atractylodes macrocephala, wheat, oats, sorghum, millet, peanuts, soybean, and Rye), add 10 mL of ultrapure water, and extract by ultrasonic treatment for 10 min. Centrifuge at 8000 rpm for 10 min. Filter the obtained supernatant using a 0.22 μm organic filter membrane to obtain the extract solution of the actual samples.

Fluorescence spectroscopy test: Add 10 μL MBI-CdSe quantum dot solution, 100 μL OTB control sample, and 100 μL actual sample extract. Vibrate the mixture to 1 mL using Tris-HCl buffer with pH = 8. After incubation in a dark chamber for 5 min, transfer to a cuvette and collect fluorescence spectra under 288 nm excitation using a fluorescence spectrometer (F-7000, Hitachi, Japan). Each sample was performed three parallel measurements.

### 2.4 Food sampling and preparation

Ten types of real food samples (rice, corn, Atractylodes macrocephala, wheat, oats, sorghum, millet, peanuts, soybean, and Rye) were collected from local markets in Hangzhou, China. Samples were randomly selected from three different batches per type to ensure representativeness. Each sample was crushed, sieved through a 50-mesh sieve, and stored in a desiccator at room temperature.

For sample extraction: accurately weigh 0.1 g of sample powder, add 10 mL of ultrapure water, ultrasonically extract for 10 min, centrifuge at 8000 rpm for 10 min, and filter the supernatant through a 0.22 μm organic filter membrane to obtain the sample extract. Finally, by diluting the OTB powder with the extraction solution, different real food samples with OTB concentrations of 1 × 10^−7^, 5 × 10^−8^, and 1 × 10^−8^ mol/L were obtained.

### 2.5 Density functional theory (DFT) calculation

The computational model was constructed using GaussView 5.0 and Gaussian 09 software. Geometric optimization and vibration frequency calculation were accomplished using density functional theory (DFT), combined with the Baker three-parameter hybrid functional and the Li-Yang-Parr correlation functional (B3LYP), using the 6-31 g(d) basis set. The B3LYP method was selected due to its higher accuracy in molecular vibration prediction and reliability, and the 6-31 g(d) basis set was chosen because of its good convergence rate of vibration frequencies.

### 2.6 Mass spectrometry analysis

The mass spectrometry adopts heated electrospray ionization source (HESI) negative ion mode with a scan range of *m*/*z* 50–750 using a Thermo Scientific Orbitrap Exploris 120 MS Mass spectrometer (USA). Gas flow rate was set as 40 mL/min, auxiliary gas flow rate was set as 10 mL/min, capillary temperature and auxiliary gas temperature was set as 350 °C. Scan mode: Primary scan resolution was 120,000 while secondary scan resolution was 1750.

## 3 Results and discussion

### 3.1 Characterization of MBI-CdSe quantum dots

**Fig. 1:**
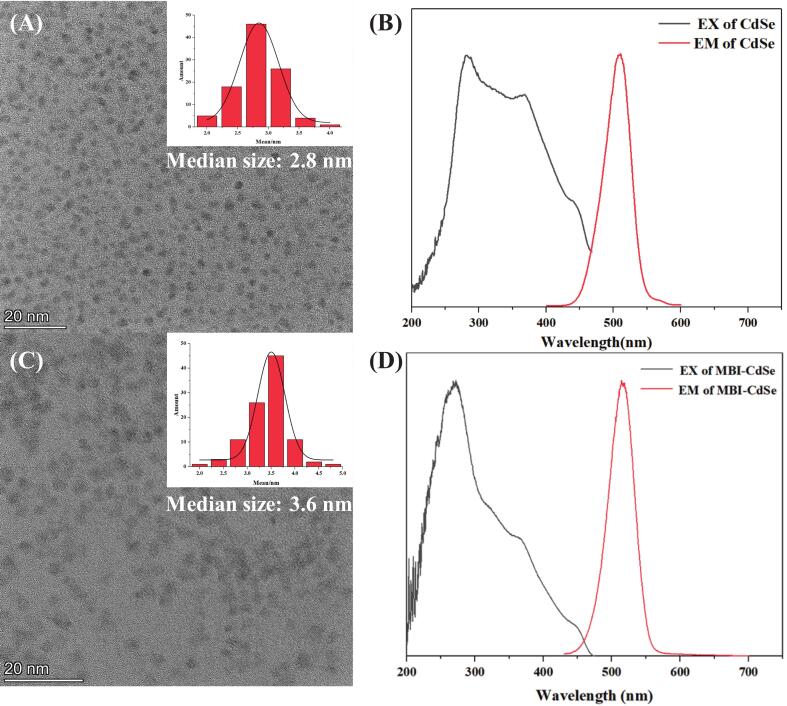
(A) TEM image of CdSe QDs, with inset showing the particle size distribution, (B) Excitation and Emission spectra of CdSe QDs, (C) TEM image of MBI-CdSe QDs, with inset showing the particle size distribution, (D) Excitation and Emission spectra of MBI-CdSe QDs.

**Fig. 2:**
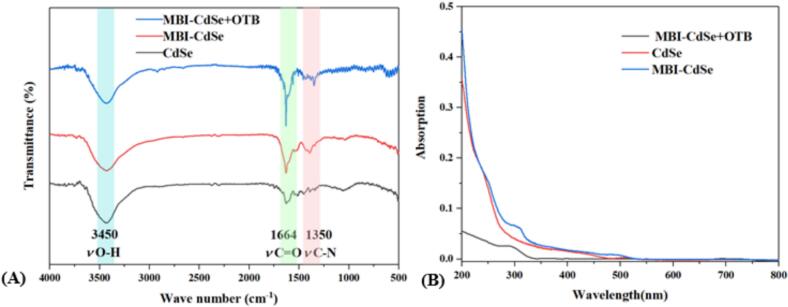
(A) IR spectra of CdSe QDs, MBI-CdSe QDs and MBI-CdSe QDs + OTB, (B) UV spectra of CdSe QDs, MBI-CdSe QDs and MBI-CdSe QDs + OTB.

The microstructure of MBI-CdSe quantum dots was first characterized using TEM. The results showed that the average particle size of CdSe quantum dots increased from 2.8 nm to 3.6 nm after MBI modification, indicating successful synthesis of MBI-CdSe quantum dots. The fluorescence excitation and emission spectra of the obtained MBI-CdSe quantum dots are shown in Fig. 1 (D). Infrared spectroscopy was used to characterize the synthesized MBI-CdSe quantum dots. As shown in Fig. 2(A), after MBI addition, two new stretching vibration peaks were observed at 1664 cm^−1^ and 1350 cm^−1^, corresponding to C=O and C—N bonds in the MBI structure. The absence of these two peaks in CdSe and the appearance of MBI-CdSe indicate that MBI has been successfully modified on the surface of CdSe. After the addition of OTB, the peak intensity has changed, which may be due to the introduction of OTB affecting the vibration absorption of hydroxyl, carbonyl, and C—N bonds. The ultraviolet spectral results (Fig. 2 (B)) also show that MBI-CdSe has a new ultraviolet absorption peak at 310 nm. Compared with MBI-CdSe and CdSe, the absorption intensity of MBI-CdSe+OTB is significantly reduced and the peak is redshifted. This is due to the electrostatic interaction between OTB and MBI-CdSe causing electron transfer. Thus, the electrostatic quenching phenomenon guided by electrostatic action is manifested.

### 3.2 Screening of optimal test conditions

The detection performance and optimal pH of MBI-CdSe quantum dots were investigated. As shown in Fig. S1, before MBI was introduced, CdSe quantum dots exhibited low pH stability and detection sensitivity, with a narrow optimal pH range and poor stability. In contrast, MBI-CdSe quantum dots demonstrated good response capability for OTB across pH values from 7 to 9. This is attributed to the imidazole group acting as an anion acceptor, which exhibits stronger binding capacity with target components under alkaline conditions. Based on this finding, Tris-HCl buffer solution with pH = 8 was selected as the optimal reaction medium in subsequent studies.

### 3.3 High sensitivity response to OTB through MBI-CdSe QDs sensor

**Fig. 3:**
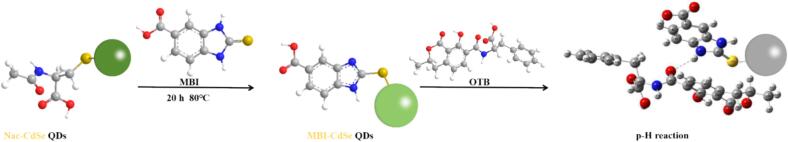
Mechanism of OTB detection by MBI-CdSe QDs and the DFT calculation results.

As shown in Fig. 3, in the 2-mercapto-benzimidazole structure, the carboxyl group introduced at the 5-position of the benzene ring enhances the electron-donating tendency of the nitrogen atom on the imidazole ring through conjugation. In the OTB structure, the isoquercenone core is linked to L-β-phenylalanine via an amide bond, forming an amide group with strong electron-withdrawing characteristics. Under alkaline conditions, this feature facilitates the formation of intermolecular hydrogen bonds between the nitrogen atom on the imidazole ring and the OTB amide group. On the other hand, the electron-withdrawing effect of the chlorine atom in the OTA structure could be transferred to the amide carbonyl group through the conjugated system, significantly reducing the electron cloud density of the carbonyl O, thereby decreasing the reactivity of the carbonyl group and limiting the ability to form hydrogen bonds with MBI. As shown in Fig. S2, the binding energy of MBI obtained from DFT calculation combined with OTB is −0.957 eV, which is much lower than +1.026 eV with OTA, and was in line with the trend of forming intermolecular single hydrogen bond binding energy. The high selectivity towards OTB was also confirmed by the appearance of a new peak at *m*/*z* 517 (MBI-OTB) in the MBI + OTB mass spectrum, while MBI + OTA did not show a new peak at the corresponding molecular weight of the bound state. The hydrogen bond length was calculated by DFT to be 2.11 Å, and the bond Angle of N-H-O was 130.55°, also verifying that the imidazolyl ligand on the surface of the quantum dot formed an intermolecular hydrogen bond with OTB. Infrared spectroscopy reveals a significant red shift in the C—N bond absorption peak of the quantum dots after OTB binding (Fig. 2(A)). Ultraviolet spectrum results (Fig. 2(B)) similarly demonstrate substantial changes in the UV absorption spectrum of the quantum dots within the 200–300 nm range following OTB addition, confirming the bond-forming interaction between MBI-CdSe quantum dots and OTB. The Zeta potential results indicated that OTB successfully bound to the imidazole group (Fig. S3). As illustrated in Fig. S4, the fluorescence lifetimes of MBI-CdSe and MBI-CdSe+OTB composite system remain essentially consistent, indicating that the OTB-induced fluorescence quenching of MBI-CdSe is static quenching. All above illustrates that the synergistic effect between the formed N-H-O intermolecular hydrogen bond and the effective electrostatic interaction formed between the positive charge on the imidazole ring and the negative charge on the carbon base in the OTB structure provides an intuitive and sensitive optical signal for the existence of OTB in food.

**Fig. 4:**
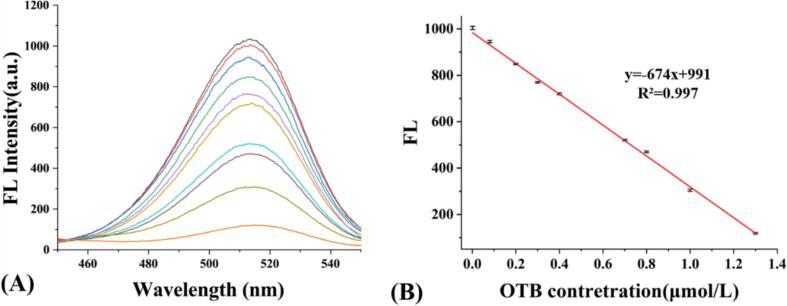
(A) Change in fluorescence intensity of MBI-CdSe QDs with increasing OTB concentration, (B)The linear regression curves of MBI-CdSe QDs with increasing OTB concentration.

The quenching effect of OTB on MBI-CdSe quantum dots exhibited a clear concentration-dependent pattern. As shown in Fig. 4, at 513 nm, the fluorescence emission intensity of MBI-CdSe quantum dots decreased with increasing OTB concentration, indicating a negative correlation between the two. A standard curve was established using OTB concentrations as the x-axis and the fluorescence intensity of MBI-CdSe quantum dots after reaction as the y-axis, yielding a linear equation y = −674× + 991 (R^2^ = 0.9970). The fluorescence intensity showed a good linear relationship with OTB concentration within the range of 10^−4^ to 0.13 μmol/L (0.037 to 48.01 ng/mL). The detection limit (LOD) reached 4.4 × 10^−5^ μmol/L (0.016 ng/mL) (LOD = 3σ/S, *n* = 3, where σ is the standard deviation of the blank solution, S is the slope of the linear fit), which is lower than the national limit standard for mycotoxins in food. Compared with traditional or similar detection techniques (Table 1), this method has superior analytical performance: not only the lower detection limit, but also a wider quantitative range, which could handle both trace sensitivity and macro accuracy. This result indicates that by introducing imidazole groups onto the surface of QDs, the intermolecular hydrogen bonds and electrostatic interactions worked synergistically, significantly enhancing the performance of the sensor.

**Table 1:** Traditional or similar OTB detection techniques.

Material	Method	Detection limit	Quantitative range
Spycata-mi3 (60-polymer Protein Nanoscaffold)	Indirect competitive enzyme-linked immunosorbent assay (ic-ELISA) (Heussner et al., 2010) (Heussner et al., 2010)	3 ng/mL	10–10,000 ng/mL
Fto-modified CdS nanorods	Photoelectrochemical(PEC)(Qileng et al., 2018)	0.04 ng/mL	0.03–10 ng/mL
Anti-OTA nanobody (Nb28)	Fluorescence resonance energy transfer(FRET)(Tang et al., 2019)	0.12 ng/mL	0.25–20 ng/mL
Colloidal gold nanoparticles (CGNs)	Colloidal gold immunochromatography test strips (CGNs strips) (Fadlalla et al., 2020)	5 ng/mL	0–10 ng/mL
This Work	Fluorescence sensors	0.016 ng/mL	0.037–48.01 ng/mL

### 3.4 Analysis of selectivity and anti-interference capability of MBI-CdSe fluorescence sensor

**Fig. 5:**
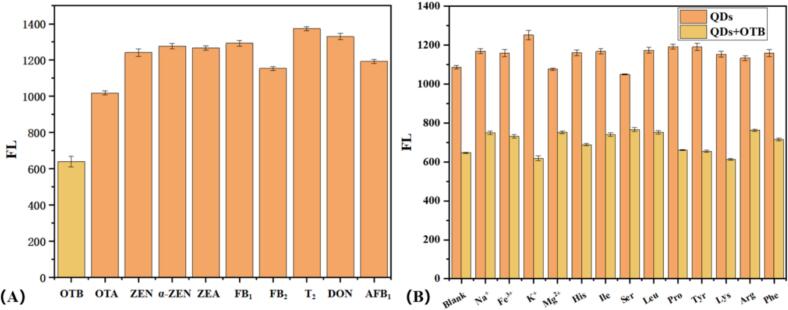
(A) Detection selectivity of MBI-CdSe QDs for OTB, (B) Anti-interference capability of MBI-CdSe QDs detection.

To evaluate the detection selectivity of MBI-CdSe quantum dots for OTB, we selected various common mycotoxins for validation. In a pH = 8 Tris-HCl buffer solution, MBI-CdSe quantum dots and mycotoxin solutions (OTB 0.5 μM while others 5 μM) were added respectively to monitor fluorescence intensity changes after mycotoxin exposure. As shown in Fig. 5(A), MBI-CdSe quantum dots exhibited significant fluorescence response only to OTB, while showing no notable quenching effect on other tested mycotoxins.

To evaluate the anti-interference performance of this sensing method, 5 μM common inorganic salts and amino acids were introduced into the detection system as potential interference sources. Fluorescence changes were compared before and after adding 0.5 μM OTB to investigate their impact on the sensor's detection capability. As shown in Fig. 5(B), the results indicate that common components such as inorganic salts and amino acids did not significantly affect the original fluorescence intensity of MBI-CdSe quantum dots or the detection sensitivity of OTB. This demonstrates that the constructed fluorescent sensor exhibits excellent anti-interference characteristics, providing strong support for its practical application in sample detection.

### 3.5 Application of MBI-CdSe fluorescence sensor in actual samples

In various matrices, the standard addition method was employed to investigate the practical application capability of MBI-CdSe quantum dots for OTB detection. The added OTB concentrations were 0.1, 0.05, and 0.01 μmol/L(36.93 ng/mL、18.47 ng/mL、3.69 ng/mL). As shown in Table 2, the detection recovery rates of OTB in 10 real food samples such as corn, rice, et al. ranged from 96.51% to 104.7%, with RSD <2.67%. This demonstrates the method's high accuracy and minimal interference from complex matrices, making it suitable for rapid quantitative detection of mycotoxins in pharmaceuticals and food products like rice, corn et al.

**Table 2:** Detection of OTB in real food samples.

Sample	Spiked concentration (μmol/L)	Predicted concentration (μmol/L)	Recovery (%)	RSD, n = 3 (%)
Rice	0.01	0.0104	104.40	1.056
	0.05	0.0485	97.05	0.453
	0.1	0.0949	96.60	2.517

Corn	0.01	0.0999	99.99	1.062
	0.05	0.0482	96.51	1.621
	0.1	0.1007	100.70	1.720

Atractylodes Lancea	0.01	0.0100	104.70	0.715
	0.05	0.0499	99.82	1.707
	0.1	0.0999	99.88	0.875

Wheat	0.01	0.0130	99.80	0.564
	0.05	0.0453	100.33	0.123
	0.1	0.1154	98.87	1.281

Oats	0.01	0.0147	99.68	0.658
	0.05	0.0426	100.52	0.353
	0.1	0.1079	99.42	0.852

Sorghum	0.01	0.0113	99.91	0.390
	0.05	0.0584	99.41	0.721
	0.1	0.1257	98.12	2.669

Millet	0.01	0.0120	99.86	0.076
	0.05	0.0537	99.74	0.610
	0.1	0.0847	101.12	1.401

Peanuts	0.01	0.0093	100.05	0.389
	0.05	0.0579	99.45	0.242
	0.1	0.1024	99.82	0.244

Soybeans	0.01	0.0143	99.71	0.932
	0.05	0.0684	98.70	0.797
	0.1	0.1027	99.80	0.304

Rye	0.01	0.0031	100.47	0.161
	0.05	0.0640	99.01	0.345
	0.1	0.1224	98.37	0.504

## 4 Conclusions

This study successfully synthesized an imidazole-modified MBI-CdSe quantum dot and developed a novel fluorescence sensing method for rapid, sensitive quantitative detection of OTB by utilizing its fluorescence quenching effect. The method exhibits a linear range of 0.037 to 48.01 ng/mL with a detection limit of 0.016 ng/mL, achieving recovery rates between 96.51% and 104.7% in real food sample testing. These results demonstrate the method's robust performance in accurately quantifying Ochratoxin B content in actual samples. Compared with other detection techniques, this method has a lower detection limit and a wider quantitative range thanks to the synergistic effect between intermolecular hydrogen bonds and electrostatic interactions which brought by the introduction of MBI onto QDs. This approach provides a novel strategy for mycotoxin detection and offers a new analytical approach for food quality evaluation.

## CRediT authorship contribution statement

**Wenxin Chen:** Writing – original draft, Data curation. **Jiahao Fu:** Writing – original draft, Validation. **Yaqi Liu:** Writing – original draft, Investigation. **Yao Fan:** Writing – review & editing, Supervision. **Haiyan Fu:** Writing – review & editing. **Yuanbin She:** Writing – review & editing, Supervision.

## Declaration of competing interest

The authors declare that they have no known competing financial interests or personal relationships that could have appeared to influence the work reported in this paper.

## Data Availability

Data will be made available on request.
