# Supplementary material for: Selective detection of Ochratoxin B in food by fluorescent sensor based on 2-mercapto-5-benzimidazole carboxylic acid-capped CdSe quantum dots
Source: Food Chem X. 2026 Mar 17;35:103770. doi: 10.1016/j.fochx.2026.103770 (PMC13015747; doi:10.1016/j.fochx.2026.103770)
Supplement: Supplementary material [file mmc1.docx]

**Selective detection of Ochratoxin B in food by fluorescent sensor based on 2-mercapto-5-benzimidazole carboxylic acid-capped CdSe quantum dots**

Wenxin Chen^1^, Jiahao Fu^1^, Yaqi Liu^1^, Yao Fan^1,*^, Haiyan Fu^2^, Yuanbin She^1,*^

^1^ *State Key Laboratory of Green Chemical Synthesis and Conversion, College of Chemical Engineering, Zhejiang University of Technology, Hangzhou 310032, PR China*

^2^ *The Modernization Engineering Technology Research Center of Ethnic Minority Medicine of Hubei Province, School of Pharmaceutical Sciences,* *South-Central University for Nationalities, Wuhan 430074, PR China*

**Corresponding Author**

^*^Yao Fan, phone: +86-0571-88320533, fax: +86-0571-88320533,

E-mail: [fany@zjut.edu.cn](mailto:fuhaiyan@mail.scuec.edu.cn)

^*^Yuanbin She, phone: +86-0571-88320533, fax: +86-0571-88320533,

E-mail: [sheyb@zjut.edu.cn](mailto:sheyb@zjut.edu.cn)


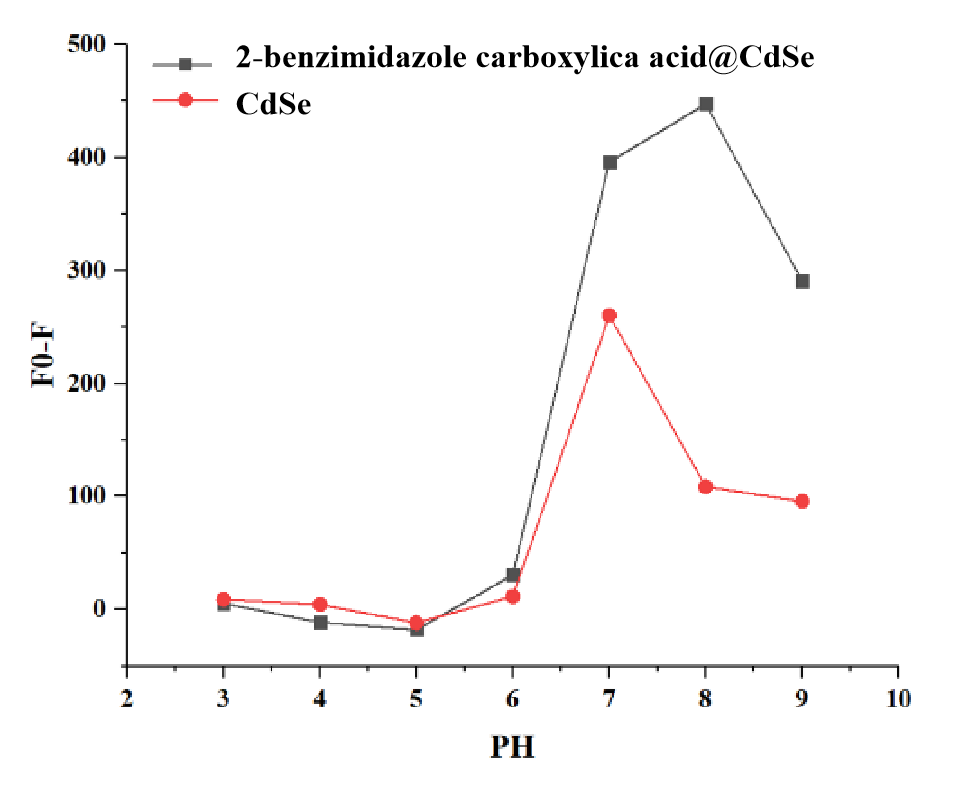


**Figure S1 Effect of pH on the Fluorescence intensity difference value of CdSe and MBI-CdSe QDs (COTB=0.25 μM)**

**
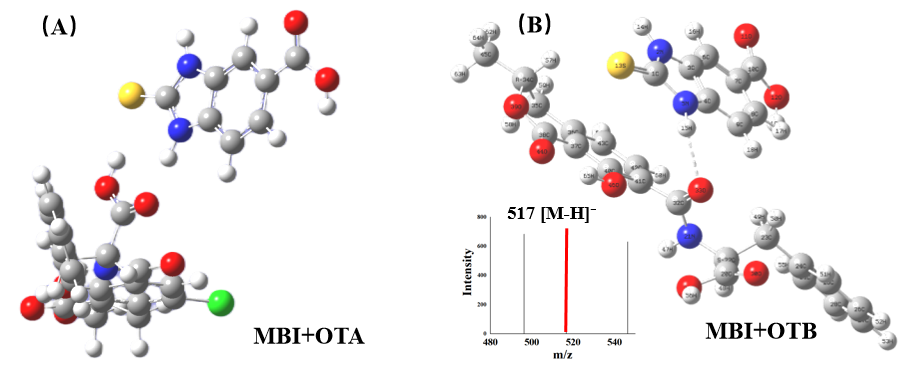
**

**Figure S2 DFT calculation results of MBI+OTA (A) and MBI+OTB (B). Inset: The mass spectrometry result of MBI+OTB.**

**
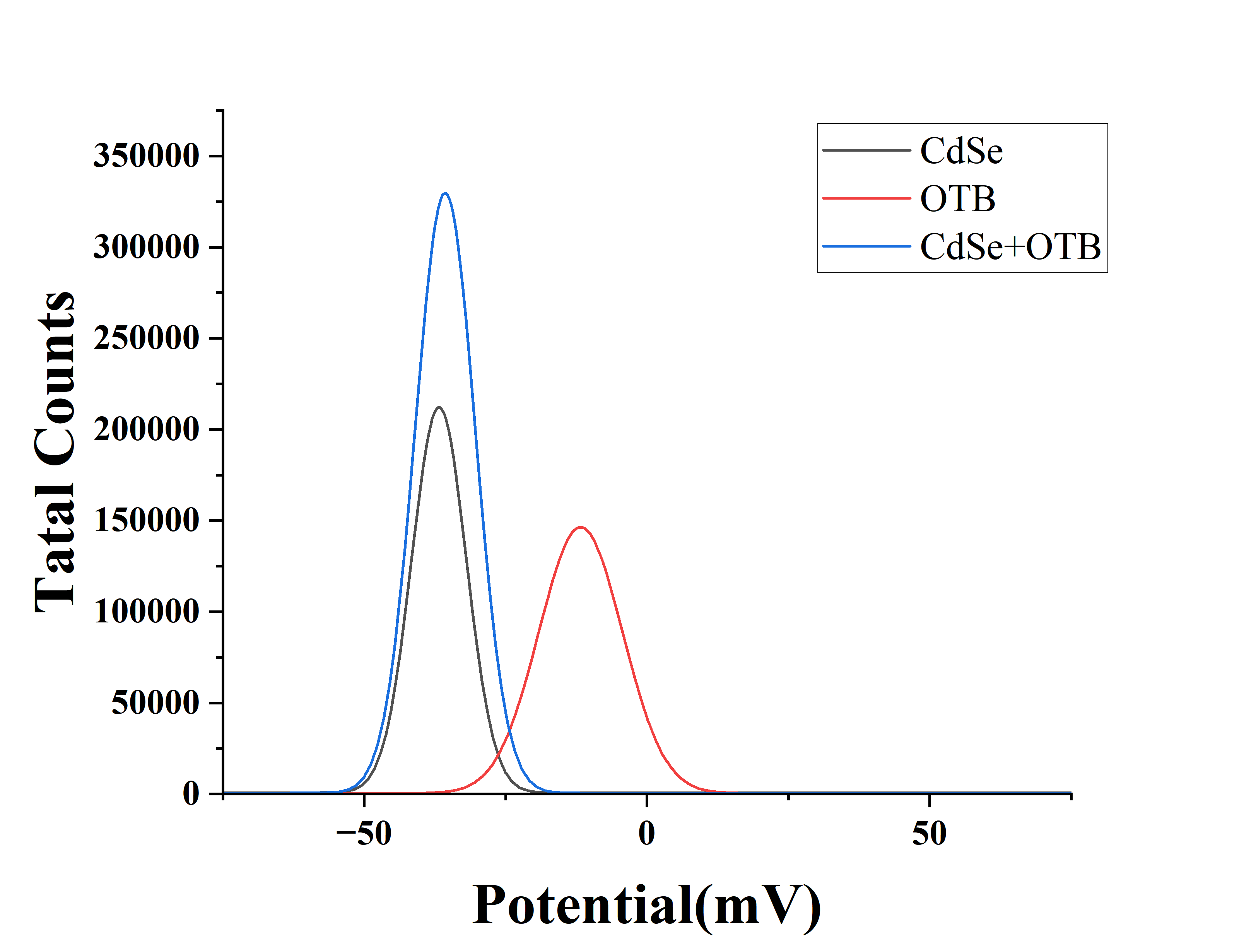
**

**Figure S3 Zeta potential diagrams of CdSe, OTB and CdSe+OTB**


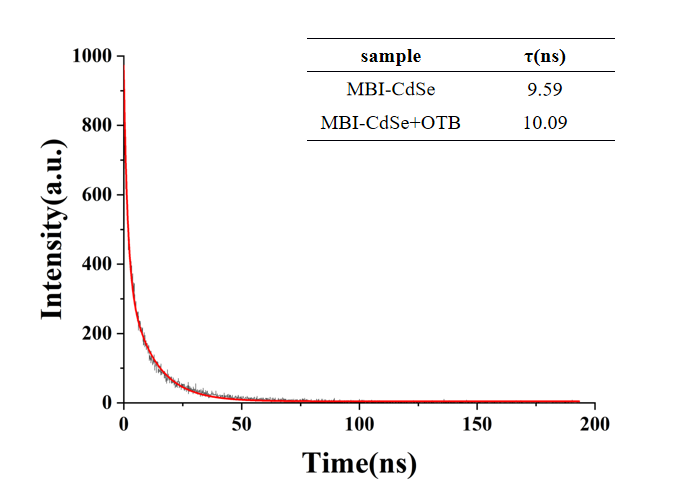


**Figure S4 The fluorescence lifetime of MBI-CdSe and MBI-CdSe+OTB**
